# Supplementary material for: Structure–Activity Relationship Target Prediction Studies of Clindamycin Derivatives with Broad-Spectrum Bacteriostatic Antibacterial Properties
Source: Molecules. 2023 Oct 31;28(21):7357. doi: 10.3390/molecules28217357 (PMC10649545; doi:10.3390/molecules28217357)
Supplement: Supplementary file 1 [file molecules-28-07357-s001.zip › molecules-2619585-supplementary.pdf]

Structure–Activity Relationship Target Prediction Studies of Clindamycin Derivatives with Broad-Spectrum Bacteriostatic Antibacterial Properties

Yiduo Jia, Yinmeng Zhang and Hong Zhu*

School of Chemical Engineering and Pharmacy, Wuhan Institute of Technology, Wuhan 430073, China

* Correspondence: 04002136@wit.edu.cn

Minimum inhibitory concentration (MIC) 3

Screening of Selumetinib targets 3

Minimum inhibitory concentration (MIC)

The MIC values of the compound were determined using the microdilution method using 96-well plates.

To determine the MIC values against gram-positive bacteria, gram-negative bacteria, and fungi, this study cultured gram-positive and gram-negative bacteria in tryptic soy broth(TSB)[14] in a 37 °C bacterial incubator for 24 h. The fungus was cultured in 50 ml TSB and bacterial culture incubator for 48 hours, the bacteria were cultured to the logarithmic stage, 100 μl of the medium was added to each well, and 1 M solution was diluted sequentially by gradient dilution. Secondly, this study sequentially adds 100 μl of bacteria or fungi to each well of the 96-well plate. Finally, this study place the 96-well plates of bacteria in a bacterial incubator at 37 °C for 24 h, while the 96-well plates of fungi are incubated in a bacterial incubator at 22 °C for 48 h. The result is shown in Table S1.

Table S1. MIC (mmol/L) of all related compounds against bacteria

Bacteria	Compound 3	Compound 3e	Compound 4
E. coli *ATCC35218*	0.0325	0.125	0.0325
K. pneumoniae *ATCC700607*	0.0325	0.125	0.0325
S. enteritidis *CICC21482*	0.0325	0.125	0.065
P. aeruginosa *ATCC27853*	0.0325	0.125	0.0325
MRSA clinical isolate	0.125	0.065	0.065
C. albicans *CMCC98001*	0.0325	0.125	0.0325

Screening of Selumetinib targets

Table S2 Targets of three compounds.

Common name					
Compound 3e		Compound 4		Compound 3	
SCN9A	ABCC1	PRKDC	ESR1	KCNH2	TTK
HTR1A	SPHK1	BACE1	ESR2	PIK3CA	DDOST
SLC6A4	MDM2	AVPR2	PAK3	ADRA1D	AURKA

CCR1	TNF	AVPR1A	XIAP	ADRA1A	SCN9A
OPRL1	ICAM1	BTK	PAK6	ADRA1B	BCL2
OPRK1	CDK9	PIK3CB	PAK4	OPRL1	TACR1
DRD4	HSP90AA1	PIK3CG	PAK2	CCR1	METAP2
HTR2A	MKNK2	PIK3CA	PAK5	PIK3CG	PDE5A
ADRA1A	MAPK14	SLC6A2	PAK1	CHRM2	IRAK1
DRD3	ITK	SLC6A4	PDE5A	CHRM1	IRAK4
KCNH2	METAP2	SLC6A3	CYP3A4	CHRM3	PDE6A
PDE4B	LTA4H	SIGMAR1	NOS2	HTR1B	MC5R
SIGMAR1	CDK2	PDE4B	ADRB2	HTR1D	MAP3K7
EGFR	CDK1	AURKB	ADRB1	HTR1A	BAD
PIK3CG	PRKDC	ALK	PIM1	SLC6A4	BCL2L2
PIK3CA	PIK3CB	RPS6KB1	PPARG	HRH1	CTRB1
IKBKB	PDE8B	AURKA	SLC5A7	CCR3	ERBB2
HRH1	PAK1	OPRK1	PIM2	ABCC1	PDE11A
CCR3	CHEK1	F10	PIM3	ROCK2	PDE4B
HTR1D	PPARA	CFD	MC4R	PIK3CD	PDE4D
ADRA1B	ADK	ADORA1	CCKBR	HTR2A	KIFC1
CXCR3	HSD11B1	ADORA2A	MC1R	DRD3	AXL

CSF1R	ADORA1	LCK	HSP90AB1	ADORA2A	TYRO3
ADRA1D	AGTR1	TNK2	MELK	TRPV3	MERTK
HTR1B	PPARG	TNF	MC3R	AKT2	PDE7A
ADRB2	SYK	BCHE	BIRC2	AKT1	PRKD1
ADRB1	TNIK	FASN	CCND3CCND1CDK4CCND2	AKT3	ABL1
ADRB3	FLT3	HRH3	MAPK14	POLR1A	RET
GABRA5	SRC	BCL2	GSK3B	HSP90AA1	ERBB4
PDE5A	PDE7A	KCNH2	PDE4D	GHSR	NAMPT
PDE6A	MAP3K14	GHSR	PRKCG	F10	PRKCB
HTR4	CFD	ACHE	PRKCD	EHMT2	SIGMAR1
AKT2	PLAT	DPP4	PRKCA	SYK	MAOA
KDR	CHRM3	ABCB1	PRKCB	HRH3	DRD4
AKT3	ADORA3	CSF1R	PRKCE	PDE8B	BTK
ERBB2	MAP2K1	FLT3	KCNA5	GABRA5	IGF1R
GNRHR	ADORA2A	HTR1A	PDGFRB	MDM2	ADRB2
ADRA2A	SPHK2	ADRA1D	AVPR1B	EGFR	ADRB1
ADRA2C	ERBB4	ADRA1A	BACE2	BCL2L1	CFD
ADRA2B	MAP3K7	ADRA1B	CCR3	ADRA2A	JAK3
HTR2C	ITGB1ITGA4	AKT1	KIT	ADRA2C	AURKB

ROCK2	KIT	KIF11	PDGFRA	ADRA2B	ADORA1
DRD1	ITGB7ITGA4	IGF1R	CHRM2	PIK3CB	SRC
F10	AURKB	ADRB3	HTR2A	MAP3K14	JAK1
HRH4	RPS6KB1	P2RX3	BCL2L1	CXCR3	JAK2
TRPV3	AURKA	CHEK1	CDK2CCNA1CCNA2	HTR4	TYK2
PIM1	HRH2	WEE1	JAK3	HTR2C	RPS6KB1
PIK3CD	PRKCB	UTS2R	JAK1	MAPK14	LTA4H
PIM2	MAPK1	FGFR1	JAK2	MAPK1	HTR2B
PIM3	CCR8	OXTR	TYK2	KDR	PIM1
